# Risk factors for excessive postoperative exo-drift after unilateral lateral rectus muscle recession and medial rectus muscle resection for intermittent exotropia

**DOI:** 10.1186/s12886-020-01484-z

**Published:** 2020-06-05

**Authors:** Shin Morisawa, Ichiro Hamasaki, Kiyo Shibata, Takehiro Shimizu, Reika Kono, Manabu Miyata, Takashi Furuse, Satoshi Hasebe, Hiroshi Ohtsuki, Yuki Morizane, Fumio Shiraga

**Affiliations:** 1 Department of Ophthalmology, Okayama University Graduate School of Medicine, Dentistry and Pharmaceutical Sciences, 2-5-1 Shikata-cho Kita-ku, Okayama, 700-8558 Japan; 2 Department of Ophthalmology and Visual Sciences, Kyoto University Graduate School of Medicine, 54 Kawahara-cho, Shogoin, Sakyo-ku Kyoto, 606-8507 Japan; 3 Department of Ophthalmology, Kawasaki Medical School General Medical Center, 2-6-1 Nakasange Kita-ku, Okayama, 700-8505 Japan; 4 Division of Ophthalmology, Okayama Saiseikai General Hospital, 2-25 Kokutai-cho Kita-ku, Okayama, 700-8511 Japan

**Keywords:** Intermittent exotropia, Postoperative exo-drift, Recurrent exotropia, Recession and resection procedure, Strabismus surgery

## Abstract

**Background:**

To detect significant factors associated with excessive postoperative exo-drift in young patients with intermittent exotropia who had undergone unilateral lateral rectus muscle recession and medial rectus muscle resection.

**Methods:**

We retrospectively examined the records of 64 consecutive patients < 18 years old who underwent surgery between April 2004 and December 2011. We sought risk factors for excessive postoperative exo-drift among patients’ demographic and clinical characteristics using univariate and multivariable linear regression analysis.

**Results:**

Younger patients (*P* = 0.007), and those with larger preoperative exo-deviation at distance (*P* = 0.033), a lower incidence of peripheral fusion at distance (*P* = 0.021) or a greater postoperative initial eso-deviation (*P* = 0.001), were significantly more likely to have an excessive postoperative exo-drift (> 20 prism diopters). Univariate analysis revealed significant associations between excessive postoperative exo-drift and age at surgery (*P* = 0.004), preoperative exo-deviation at distance (*P* = 0.017) and postoperative initial eso-deviation at distance (*P* < 0.001). Multivariable linear regression analysis showed that postoperative initial eso-deviation at distance (*P* = 0.008) was significantly associated with postoperative exo-drift.

**Conclusions:**

Postoperative exodrift in unilateral RR is predicted by the initial postoperative eso-deviation, which may offset the overcorrection. However, the exo-drift is greater in cases with a large preoperative exo-deviation and/or at a younger age, and should be followed carefully.

## Background

In the surgical treatment of intermittent exotropia, most clinicians aim to achieve overcorrection at the initial postoperative examination [1, 2]. Exotropia may recur gradually over months or years after surgery, a phenomenon known as postoperative exo-drift [3]. Ideally subsequent postoperative exo-drift should cancel out any overcorrection, but unexpectedly large postoperative exo-drift can result in recurrent exotropia. Excessive postoperative exo-drift diminishes the long-term surgical success rate, and makes it difficult to compare the findings of studies in which outcomes were recorded at different follow-up period. A better understanding of excessive exo-drift, the risk factors and means of preventing it are needed. In many studies of intermittent exotropia, patients had undergone a variety of procedures, including bilateral lateral rectus muscle recession (BLR), unilateral recession and resection (RR) or unilateral lateral rectus muscle recession (ULR), making it difficult to interpret the findings due to the potential influence of surgical technique on exo-drift [4–7]. We examined the factors associated with postoperative exo-drift in young patients with intermittent exotropia who had undergone only unilateral RR to establish risk factors for recurrent exotropia.

## Methods

The records of a series of 64 consecutive patients aged < 18 years with intermittent exotropia who underwent unilateral RR surgery between April 2004 and December 2011 at Okayama University Hospital were examined retrospectively. Subjects were 31 males (48%) and 33 females (52%). Operated eyes were 29 right (45%) and 35 left (55%). We excluded the following example: preoperative vertical deviation of > 5 prism diopters (PD), dissociated vertical deviation, previous strabismus surgery, surgery with vertical transposition, other disease causing ocular deviation (for example, thyroid ophthalmopathy, myasthenia gravis, internuclear ophthalmoplegia, high grade myopia, orbital dysplasia, paretic strabismus, sensory strabismus or other neurologic disorders).

We recorded age at surgery, preoperative angle of deviation at distance (5 m) (a negative value indicating exo-deviation, and a positive value eso-deviation), preoperative near-distance disparity in angle of deviation (by subtracting distance angle of deviation from near (33 cm) angle of deviation; positive value indicating convergence insufficiency), the refractive error in the operative eye, the difference between the refractive error of both eyes, the difference between the visual acuity of both eyes using the logarithm of minimum angle of resolution (logMAR), stereoacuity threshold using the TNO test (Ootech, AG Veeneldaal, Netherlands) transformed to log seconds of arc (arcsec), the presence or absence of peripheral fusion at distance and near (assigned a value of 1 or 0, respectively and assessed using the Bagolini striated glass test), the postoperative initial angle of deviation at distance, the postoperative initial near-distance disparity and last postoperative angle of deviation. The Shapiro-Wilk Test was used to assess data for normality. The stereoacuity threshold was 1980 arcsec (range 15 arcsec to 33 arcmin) measured using the TNO test. Absence of stereopsis using the TNO was assigned a value of the next level to 66 arcmin. The assignment of the next log level is commonly used in analysis of stereoacuity data and allows for calculations of changes in stereoacuity.

**Fig. 1:**
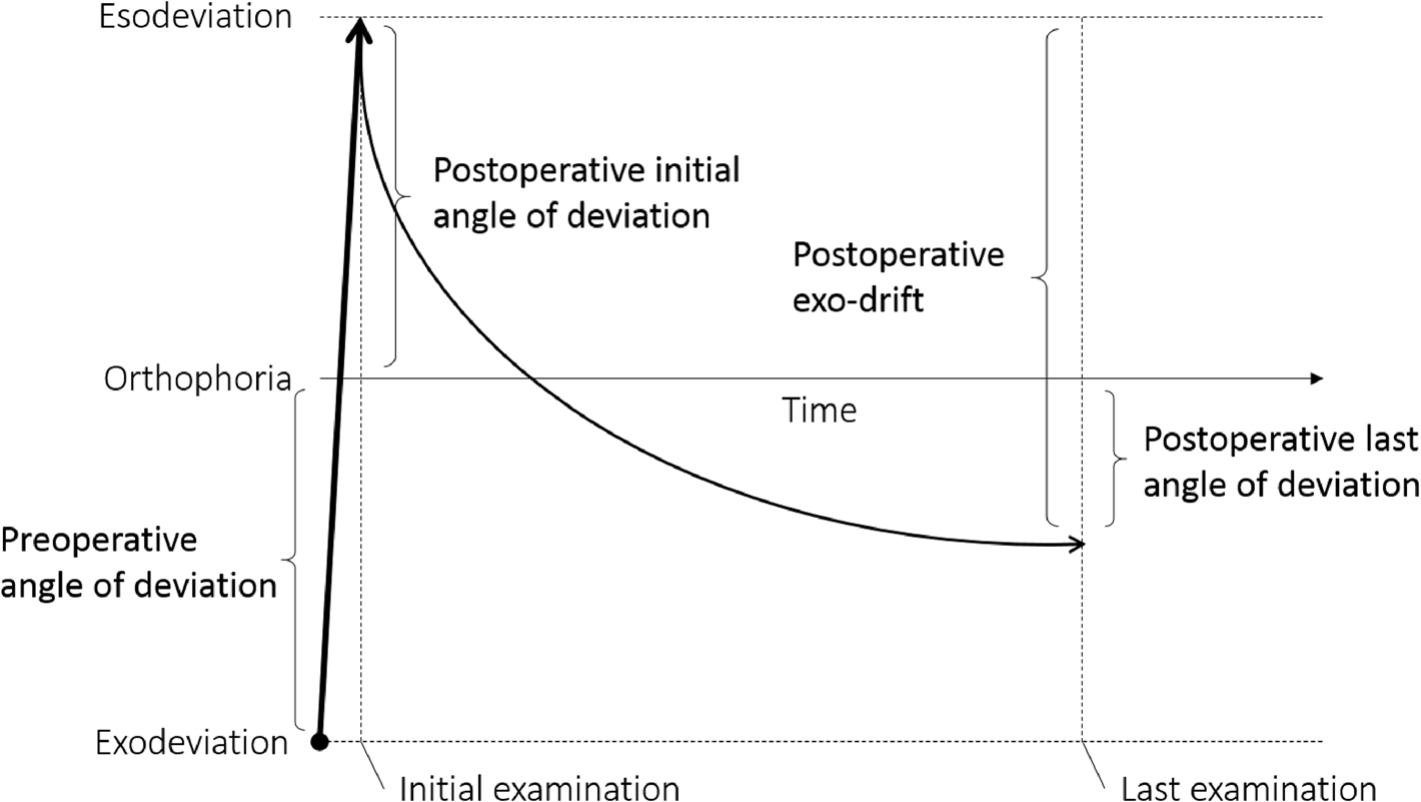
Definitions of outcome measures. Horizontal axis, time; vertical axis, angle of deviation; bold arrow, surgery

The extent of preoperative angle of exodeviation at distance fixation was recorded in each subject by means of the PAT, using the Fresnel Press-On Prism (Health Care Specialties Division/3 M; St. Paul, MN, USA), which was attached to glasses at two equal parts of the PD to neutralize the angle of deviation. The PD was adjusted according to responses to deviation as determined by the prism and cover test (PCT), and the test was repeated at 20-min intervals until no additional prisms were required to neutralize the distance deviation. The amount of surgery was determined by measurements at distance fixation [8]. Preoperatively, the hole-in-the-card test was performed to determine the dominant eye. The eye the patient used to view the target through the hole was defined as the dominant eye. Surgery was performed on the nondominant eye. The amount of surgery was based on the smallest angle of deviation at distance or near fixation. In all cases, the same amount (1 mm per 5PD) of lateral rectus muscle recession and medial rectus muscle resection was carried out, referring to the strabismus surgical amount table of Okayama University Hospital. The alternative prism cover test was used to measure angle of deviation approximately 1 week and 1 year after surgery due to the small residual angle of deviation [9]. The difference between the angle of deviation recorded at the initial examination and that recorded at the last examination was defined as postoperative exo-drift (Fig. 1).

Patients were divided into two groups according to the extent of postoperative exo-drift: those with excessive postoperative exo-drift > 20 PD were allocated to group A; those with postoperative exo-drift ≤20 PD to group B. Data are presented as mean ± SD unless otherwise stated. The Mann–Whitney U test was used to test for significant differences between the groups. Correlation analyses were used to assess the strength of the association between each pre-drift parameter and postoperative exo-drift and expressed as the Spearman rank-correlation. These findings were used to inform subsequent multivariable linear regression analysis using a direct entry method. We used IBM SPSS Statistics for Windows, Version 22.0 (IBM. Corp., Armonk, NY, USA) for all statistical analyses.

## Results

The mean age at surgery was 9.4 (±3.5) years (range: 5–17 years); patients’ pre-drift parameters are shown in Table 1. The mean time elapsed to the first postoperative examination was 6.2 (±1.7) days (range: 1–13 days) and to the last examination was 650 (±195) days (range: 295–1153 days). Postoperative elapsed time to the last examination did not significantly relate to postoperative exo-drift and correlation coefficient was − 0.124 (*P* = 0.329). The mean last postoperative angle of deviation at distance was − 5.0 (±4.9)° (range: − 16.7–9.1°): a negative value indicating exotropia. Mean post-operative exo-drift was − 12.2 ± 4.6° (range: − 23.1– -3.4°). None of the parameters were normally distributed, therefore relationships between the parameters were assessed using Spearman rank-correlation.

**Table 1:** Subjects’ summary in pre-drift parameters

Amount of recession / resection (SD) (range)	6.4 (1.3) mm (4.0–9.0)
Preoperative angle of deviation at distance (SD) (range)	−17.5 (3.7)° (− 27.6– − 10.3).
Preoperative near-distance disparity (SD) (range)	2.2 (3.3)° (−5.7–11.0)
Refractive error in the operative eye (SD) (range)	− 1.1 (2.1) diopters (− 10.1–5.9)
Difference between refractive error of both eyes (SD) (range)	0.5 (0.8) diopters (0.0–4.4)
Difference between visual acuity of both eyes (SD) (range)	0.0 (0.1) (0.0–0.2)
Stereoacuity threshold transformed to log (SD) (range)	2.0 (0.6) log arcsec (1.2–3.6)
Peripheral fusion at distance fixation (proportion)	22 (34%)
Peripheral fusion at near fixation (proportion)	48 (75%)
Initial postoperative angle of deviation at distance (SD) (range)	7.3 (5.2)° (−2.9–21.8)
Initial postoperative near-distance disparity (SD)	1.8 (4.3)° (−11.9–10.8)

Characteristics of patients with postoperative exo-drift > 20 PD and ≤ 20 PD are shown in Table 2. Those with excessive postoperative exo-drift (Group A) were significantly younger at surgery, had greater preoperative exo-deviation, a lower incidence of peripheral fusion, greater overcorrection at the initial postoperative examination and larger last postoperative exo-deviation than those with less postoperative exo-drift (Group B).

**Table 2:** Characteristics of patients with postoperative exo-drift > 20 PD and ≤ 20 PD.

Parameter	Group A (*n* = 36)	Group B (*n* = 28)	*P* value
**Age at surgery**	**8.4 ± 2.8**	**10.8 ± 3.8**	0.007 *
**Preoperative angle of deviation at distance**	**−18.3 ± 3.8°**	**−16.4 ± 3.2°**	0.033 *
Preoperative near-distance disparity in deviation	1.5 ± 3.4°	3.1 ± 3.1°	0.088
Refractive error in the operative eye	−0.8 ± 1.5	−1.3 ± 2.8	0.091
Difference between refractive error of both eyes	0.4 ± 0.5	0.7 ± 1.1	0.113
Difference between visual acuity of both eyes	0.027 ± 0.041	0.045 ± 0.060	0.257
Stereoacuity values transformed to log arcsec	2.1 ± 0.6	2.0 ± 0.6	0.799
**Peripheral fusion at distance**	**22 ± 42%**	**50 ± 51%**	0.021 *
Peripheral fusion at near	78 ± 42%	71 ± 46%	0.564
**Initial postoperative angle of deviation at distance**	**9.3 ± 5.1°**	**4.7 ± 4.0°**	0.001 *
Initial postoperative near-distance disparity in deviation	2.1 ± 4.5°	1.4 ± 4.1°	0.357
**Last postoperative angle of deviation at distance**	**−6.3 ± 5.5°**	**−3.4 ± 3.5°**	0.017 *
Postoperative elapsed time to the last examination	660 ± 199 days	636 ± 193 days	0.756

All data are presented as mean ± standard deviation

* represents statistical significance (*P* < 0.05)

On correlation analysis, relationships between clinical characteristics and postoperative exo-drift are shown in Table 3. Greater postoperative exo-drift was associated with younger age at surgery, larger preoperative exo-deviation at distance and greater initial postoperative eso-deviation at distance.

**Table 3:** Relationships between clinical characteristics and postoperative exo-drift

Pre-drift parameter	Correlation coefficient	*P* value
**Age at surgery**	**0.357**	0.004 *
**Preoperative angle of deviation at distance**	**0.296**	0.017 *
Preoperative near-distance disparity in deviation	0.240	0.056
Refractive error in the operative eye	−0.191	0.130
Difference between refractive error of both eyes	0.237	0.059
Difference between visual acuity of both eyes	0.223	0.076
Stereoacuity transformed to log arcsec	−0.005	0.971
Peripheral fusion at distance	−0.066	0.604
Peripheral fusion at near	0.064	0.613
**Initial postoperative angle of deviation at distance**	**−0.560**	< 0.001 *
Initial postoperative near-distance disparity in deviation	−0.139	0.275

*represents statistical significance (*P* < 0.05)

Multiple linear regression analysis was also performed. Postoperative exo-drift was defined as the dependent variable, and other pre-drift parameters were defined as the independent variables. The only significantly influential factor was initial postoperative angle of deviation at distance (*P* = 0.008, Table 4).

**Table 4:** Multivariable linear regression analysis using a direct entry method

Coefficient of determination in this model	0.426		
*P*-value in analysis of variance in this model	0.001		
Pre-drift parameter	Unstandardized coefficients	Standardized coefficients	*P* value
Age at surgery	− 0.378	0.282	0.055
Preoperative angle of deviation at distance	0.238	0.188	0.116
Preoperative near-distance disparity in deviation	0.098	0.070	0.614
Refractive error in the operative eye	−0.171	−0.079	0.543
Difference between refractive error of both eyes	0.162	0.029	0.830
Difference between visual acuity of both eyes	18.3	0.200	0.177
Stereoacuity values transformed to log arcsec	0.197	0.024	0.860
Peripheral fusion at distance	0.700	0.076	0.498
Peripheral fusion at near	0.819	0.062	0.634
**Initial postoperative angle of deviation at distance**	**−0.311**	**−0.347**	0.008 *
Initial postoperative near-distance disparity in deviation	−0.268	− 0.248	0.070
Constant	−10.9		0.040

* represents statistical significance (*P* < 0.05)

## Discussion

Age at surgery correlated with postoperative exo-drift in our cohort, with younger patients more likely to develop greater exo-drift. Yam and colleagues reported that a non-significant trend suggestive that age at surgery influenced exo-drift in patients undergoing BLR because their report limited the age to 96.5 ± 43.8 months [10]. However, range of age at surgery was more variable in this study. We consider age at surgery to be a key preoperative influencer of postoperative exo-drift, likely because of degeneration of orbital connective tissue that effects ocular alignment with aging [11, 12].

Age at surgery has been reported not to influence final outcome after RR surgery in the short-, medium- or long-term in some previous reports [13, 14]. In our cohort, age at surgery correlated with postoperative initial angle of deviation, with the most extensive eso-deviation seen in younger patients: the younger the age at surgery, the larger the exo-drift and eso-deviation in the initial postoperative examination. Thereafter, compensating exo-drift may mean that the difference in postoperative deviation at initial examination between younger and older ages may become weak or absent in the longer term.

Both univariate and multivariable analysis identified initial postoperative angle of deviation at distance as being significantly associated with postoperative exo-drift. In addition, the initial overcorrection was significantly greater in those with excessive postoperative exo-drift > 20 PD than those with exo-drift ≤20 PD, a relationship also reported by Yam and colleagues [10]. The greater the overcorrection after surgery, the larger the exo-drift. Exo-drift may therefore balance out overcorrection, a hypothesis confirmed by reports that initial postoperative angle of deviation is not associated with angle of deviation 1 year or more after surgery [10, 15–17]. This also agrees with Park and colleagues’ report that the rate of exo-drift is greater in those with more extensive overcorrection immediately after surgery [18], and a report that surgical outcome is not significantly different between traditional BLR and a surgical technique modified by reducing the amount of resection by 1–2 mm [19].

Preoperative angle of exo-deviation is reportedly associated with postoperative exo-drift in patients who underwent BLR [10, 17]. We also detected this relationship in our patients: more extensive preoperative exo-deviation appeared to predict more extensive postoperative exo-drift. In addition, the preoperative angle of exo-deviation was greater in those with excessive postoperative exo-drift (Group A) than those with postoperative exo-drift ≤20 PD (Group B). Surgeons should consider the potential for postoperative exo-drift to result in excessive exo-deviation in each case of RR or BLR.

We found that those with postoperative exo-drift >20PD had greater last postoperative exo-deviation despite an initially larger initial postoperative eso-deviation than those with postoperative exo-drift ≤20 PD. An unexpectedly large postoperative exo-drift is an important risk factor for recurrent exotropia. In consideration of comparing between two groups, a lower incidence of peripheral fusion at distance might have been expected to influence the extent of postoperative exo-drift, but we found no significant relationship in either our univariate or multivariable analyses.

It is difficult to compare our findings with those of other investigators due to the possibility that surgical approach influenced the extent of exo-drift [4–7], although there have been reports that surgical technique is not a significant risk factor for exo-drift [13, 18, 20]. The influence of surgical technique on exo-drift remains a matter of considerable debate. Intermittent exotropia associated with A and V patterns is also reportedly associated with less postoperative exo-drift [21], but these patients were excluded from our analysis.

In addition, last postoperative examinations were approximate 1 year or later and variety in this retrospective study. It has little effect on our results because postoperative exo-drift is considered to be stable after postoperative 1 year [22]. Because of significant difference in the amount of postoperative exo-drift by age, this study has the advantage of limiting the age to less than 18 years. Cases were limited to unilateral RR. Accordingly, the number of cases has been limited. This study does not include information on the amount of time participants had a manifest deviation. Therefore, the level of control of their deviation cannot be evaluated. In the Bagolini striated glass test in this study, the sensory fusion and motor fusion could not be separated because the prism was not used to correct the eye position.

## Conclusions

We found that in our cohort of young patients undergoing unilateral RR for intermittent exotropia, younger age at surgery, greater preoperative exo-deviation and greater postoperative initial eso-deviation were significantly associated with greater postoperative exo-drift. Postoperative exo-drift in unilateral RR is predicted by the initial postoperative eso-deviation at a distance, which may offset the overcorrection. However, the exo-drift is greater in cases with a large preoperative exo-deviation at a distance and/or at a younger age, and should be followed carefully. Our findings will help for predicting and evaluating postoperative exo-drift.

## Data Availability

The datasets used and/or analysed during the current study are available from the corresponding author on reasonable request.

## Abbreviations

BLR: Bilateral lateral rectus muscle recession
RR: Recession–resection
ULR: Unilateral lateral rectus muscle recession
PD: Prism diopters
PAT: Prism adaptation test
SD: Standard deviations
arcsec: Arc second
arcmin: Arc minute
